# Chloroform Inhalation—Danger from Chloroforrm

**Published:** 1871-02

**Authors:** B. W. Richardson

**Affiliations:** Braithwaite


### Chloroform Inhalation.—Danger from Chloroforrm.

The only condition of body which may be diagnosed as especially dangerous for chloroform is that of a weakened and dilated right side of the heart, with enlarged hemorrhoidal veins, varicose veins, of the lower extremities, and large, full, yet not tense veins in the other parts of the body. Beyond this our knowledge ceases, for nothing definite is known.

*Prevention of Danger from Chloroform.*—Be sure that the breathing is unimpeded, and that no weight of bed-clothes rests on the abdominal muscles. The sitting posture is unfavorable for the heart, and the lying position for the respiration. On all accounts the semi-recumbent position is the best, as it is generally the most convenient. It is well to keep the body from the beginning to the end of the operation in the same position, for, in experiments on animals it is found that they may be narcotised until the inspiratory act ceases, and yet the animal may recover; but at this crisis the smallest movement, the merest handling of the body will prevent all chance of return of power. Although chloroform should not be administered to a patient whose stomach is charged with food, it is very bad practice to allow the system to become exhausted for want of food before the chloroform is given.

*Death from Chloroform.*—If a warm-blooded animal is subjected suddenly to the vapor of chloroform, at a temperature over 70 deg. Fahr., it will often cease to breathe and to circulate blood at once. Again, it is found that a considerable proportion of deaths from chloroform occur within the first minute of its administration. These things show us that it is a bad practice to commence its inhalation too abruptly, or to force on narcotism rudely, against time. The same observation extends to all bodies of the same family, as chloride of methyl, and bichloride of methylene. In these cases it is not the quantity of the vapor absorbed into the blood which kills: the killing is by a primary impression on the peripheral nervous surface from the vapor, and by consequent arrest of the action of the heart from asphyxia of the blood. Various other well ascertained facts prove that the best plan of giving chloroform is to carefully feel the way in the first minute or two of administration, and then, in the adult to give it freely so as to push quickly into the third degree of anæsthesia.

There are four modes of death from chloroform. In the first, by the immediate influence exerted by the chloroform on the peripheral nervous system respiration is for an interval suspended, there is accumulation of carbonic acid in the blood, irritation of the vagus, and consequent arrest of the action of the heart. Artificial respiration offers the best chance of recovery in this form of death, because the irritability of the heart is unimpaired. Nervous irritable people are those subject to this. The second mode of death may be called *epilepti from syncope;* it is instantaneous, and we find the arteries completely empty of blood, and the brain blanched and bloodless. This form of death occurs during the second stage, or that of excitement. The third form of death occurs when, from the slow and continued action of the narcotic, there is *paralysis of the heart.* This form of death is hopeless, artificial respiration has no effect on it. It is always preceded by intermittent action of the heart. The fourth form of death is a compound one—there is first depression of the heart and system from the chloroform, and then surgical shock is superadded. Hemorrhage may have aided the dedepression of system. Death here is by syncope, and is often sudden. It is very liable to occur from committing the error of supposing that in small operations it is only necessary to administer a little narcotic vapor, and, secondly, from proceeding to operate while the patient is excited and not insensible. The best means of producing artificial respiration is by Richardson's double-acting bellows.—*Dr. B. W. Richardson, in Braithwaite's.*

